# Genetic diversity and structure in *Arapaima gigas* populations from Amazon and Araguaia-Tocantins river basins

**DOI:** 10.1186/s12863-018-0711-y

**Published:** 2019-01-28

**Authors:** Lucas Simon Torati, John Bernard Taggart, Eduardo Sousa Varela, Juliana Araripe, Stefanie Wehner, Hervé Migaud

**Affiliations:** 1 EMBRAPA Pesca e Aquicultura, Palmas, TO CEP 77008-900 Brazil; 2 Institute of Aquaculture, University of Stirling, Stirling, FK9 4LA Scotland, UK; 3 Instituto de Estudos Costeiros, Campus de Bragança, Universidade Federal do Pará, Bragança, PA CEP 68600-000 Brazil; 4 Max Planck Institute of Psychiatry, Kraepelinstr. 2-10, 80804 Munich, Germany

**Keywords:** Aquaculture, Conservation, ddRAD, Fisheries, Genetics, Pirarucu

## Abstract

**Background:**

*Arapaima gigas* (Schinz, 1822) is the largest freshwater scaled fish in the world, and an emerging species for tropical aquaculture development. Conservation of the species, and the expansion of aquaculture requires the development of genetic tools to study polymorphism, differentiation, and stock structure. This study aimed to investigate genomic polymorphism through ddRAD sequencing, in order to identify a panel of single nucleotide polymorphisms (SNPs) and to simultaneously assess genetic diversity and structure in wild (from rivers Amazon, Solimões, Tocantins and Araguaia) and captive populations.

**Results:**

Compared to many other teleosts, the degree of polymorphism in *A. gigas* was low with only 2.3% of identified RAD-tags (135 bases long) containing SNPs. A panel of 393 informative SNPs was identified and screened across the five populations. Higher genetic diversity indices (number of polymorphic loci and private alleles, Shannon’s Index and H_O_) were found in populations from the Amazon and Solimões, intermediate levels in Tocantins and Captive, and very low levels in the Araguaia population. These results likely reflect larger population sizes from less urbanized environments in the Amazon basin compared to Araguaia. Populations were significantly differentiated with pairwise *F*_ST_ values ranging from 0.086 (Amazon × Solimões) to 0.556 (Amazon × Araguaia). Mean pairwise relatedness among individuals was significant in all populations (*P* < 0.01), reflecting a degree of inbreeding possibly due to severe depletion of natural stocks, the species sedentary behaviour and possible sampling biases. Although Mantel test was not significant (*P* = 0.104; R^2^ = 0.65), Bayesian analysis in STRUCTURE and discriminant analysis of principal components (DAPC) showed populations of Amazon and Solimões to be genetically differentiated from Araguaia, with Tocantins comprising individuals from both identified stocks.

**Conclusions:**

This relatively rapid genotyping by sequencing approach proved to be successful in delineating arapaima stocks. The approach and / or SNP panels identified should prove valuable for more detailed genetic studies of arapaima populations, including the elucidation of the genetic status of described discrete morphotypes and aid in delivery of conservation programs to maintain genetic diversity in reservoirs across the Amazon region.

**Electronic supplementary material:**

The online version of this article (10.1186/s12863-018-0711-y) contains supplementary material, which is available to authorized users.

## Background

Over the past 10–15 years the detection and analysis of genome-wide single nucleotide polymorphism (SNP) markers have revolutionized genetic investigations of all types of organisms. As elsewhere, within the field of fisheries science, SNPs are becoming the marker of choice for population genetic studies [1, 2], their abundance, distribution and relative ease and accuracy of scoring also proving invaluable in other research areas e.g. elucidating sex determination systems [3–5]; constructing genetic linkage maps [6–8] and aiding whole genome selection for many farmed species [5, 9]. The bi-allelic nature of SNPs, which allows more confident estimation of allelic frequencies from small sample sizes, and the ability to survey both neutral and non-neutral loci, presents the opportunity to explore different and complementary perspectives cf. microsatellite based population genetic and conservation studies [10–17].

Genotype by sequencing approaches, including restriction-site-associated DNA (RAD) [18] and double digest RAD (ddRAD) [19], provide a scalable, rapid and relatively inexpensive means to simultaneously discover and genotype hundreds to thousands of SNPs in non-model species. These methodologies do, however, require empirical optimization as the number of loci detected is largely dependent on genome size, architecture and base content of the species under study, while the extent of SNP polymorphism can also vary significantly.

The Amazonian pirarucu *Arapaima gigas* (Schinz, 1822) is the largest freshwater scaled fish in the world with adults reaching up to 250 kg and measuring over 2.5 m in total length [20]. This emblematic air-breather fish is a promising candidate species for aquaculture development and has a valuable market in South America [21]. The natural geographical distribution of *A. gigas* includes the basins of the Amazon, Tocantins-Araguaia and Essequibo rivers, which cover Brazil, Ecuador, Guyana and Peru. Also, the species has already been introduced into several non-indigenous water systems [22]. *Arapaima gigas* is a dioecious and iteroparous species with sexual maturity reached after 3 to 5 years of age [23]. Reproduction involves nest building by males and females in the sandy bottom of lentic habitats during the rainy season from November onwards [24, 25]. External fertilisation generally involves a single female, often with contributions from more than one male, a strategy that helps maintain genetic diversity in the species [26]. After spawning, the nest is guarded by both parents until egg hatching, then parental care is provided by the male and a characteristic lateral migration towards flooded food-rich areas ensues [25]. Females normally reproduce multiple times within the reproductive season [23] with a mean fecundity estimated to be c. 11,000 fingerlings counted at the parental care phase (up to 3 months post-hatching) per spawning event, and a balanced sex ratio at hatch [27, 28]. During the dry season, from June onward, water levels in the rivers decrease, marking the end of the parental care [25]. At this stage, adults and fingerlings migrate back to the low lands and disperse in the river canals and floodplain lagoons [25, 29]. Overall, adults are not believed to migrate long distances. They are solitary and well adapted to hypoxic conditions during the drought season [25, 29]. In some regions and years the dry season can be severe, resulting in mass mortalities of *A. gigas* and rescuing operations are organised for conservation reasons [30].

Given its obligate air-breathing behaviour, *A. gigas* is an easy target for fishermen and natural populations have been historically depleted or even eradicated close to main cities [31]. It is estimated that populations today represent only 13% of historic levels [32] and since 1986 *A. gigas* has been included in the CITES red list [22]. While occasionally successful, breeding of *A. gigas* in captivity is not a routine practice due to complex reproductive traits and dysfunctions in the species (i.e. failure at the final oocyte maturation and ovulation stage, lack of male-female synchronization at spawning), which require further research especially for gender identification and control of spawning [33, 34]. Therefore, fingerlings are valuable in the aquaculture market, and their illegal capture from the wild is a challenge for conservation. Translocations of animals are also a concern as morphotypes (white vs orange fleshed individuals) [31] and potentially novel species have been described, suggesting patterns of allopatric differentiation across different hydrographic basins [35–37].

To date, limited numbers of studies have been conducted to characterise the genetic diversity and structure of natural *A. gigas* populations. These have involved the use of mitochondrial markers (mtDNA), microsatellite or inter-simple sequence repeats (ISSR) markers to study eight populations from the Amazon, Solimões and Tocantins river systems [29, 31, 38, 39], four [30] and five [40] populations from Tocantins and Araguaia and five populations from Essequibo and Branco rivers [37]. Overall, these studies found higher levels of genetic diversity within the large Amazon River basin compared to other systems with the population structure suggesting minimal genetic flow and high genetic differentiation between populations. So far, molecular data has failed to confirm a multispecies scenario for *Arapaima*, which today is supported only by morphological analyses of very few specimens [35–37]. Further genetic research on *A. gigas* has focused on acquiring tools for gender identification since this species is not sexually dimorphic, a factor that has impeded captive reproduction and aquaculture development. To do so, the species karyotype was characterised (2n = 56) but no apparent sex chromosome dimorphism was observed [41, 42], while later a bulked segregant analysis failed to find sex-related markers [43]. Next generation sequencing (NGS) technologies have not yet been applied to investigate genomic variability and diversity in populations of *A. gigas*.

In the current study the potential of ddRAD to generate an informative SNP panel for population genetic analyses of the endangered *A. gigas* was explored. Genetic diversity and structure among five population samples (four wild samples from different river systems, one captive stock) was assessed and compared with published studies using microsatellite and mtDNA markers. The relevance of the results for future conservation and exploitation of this iconic species is discussed.

## Results

### ddRAD sequencing and degree of polymorphism

**Fig. 1:**
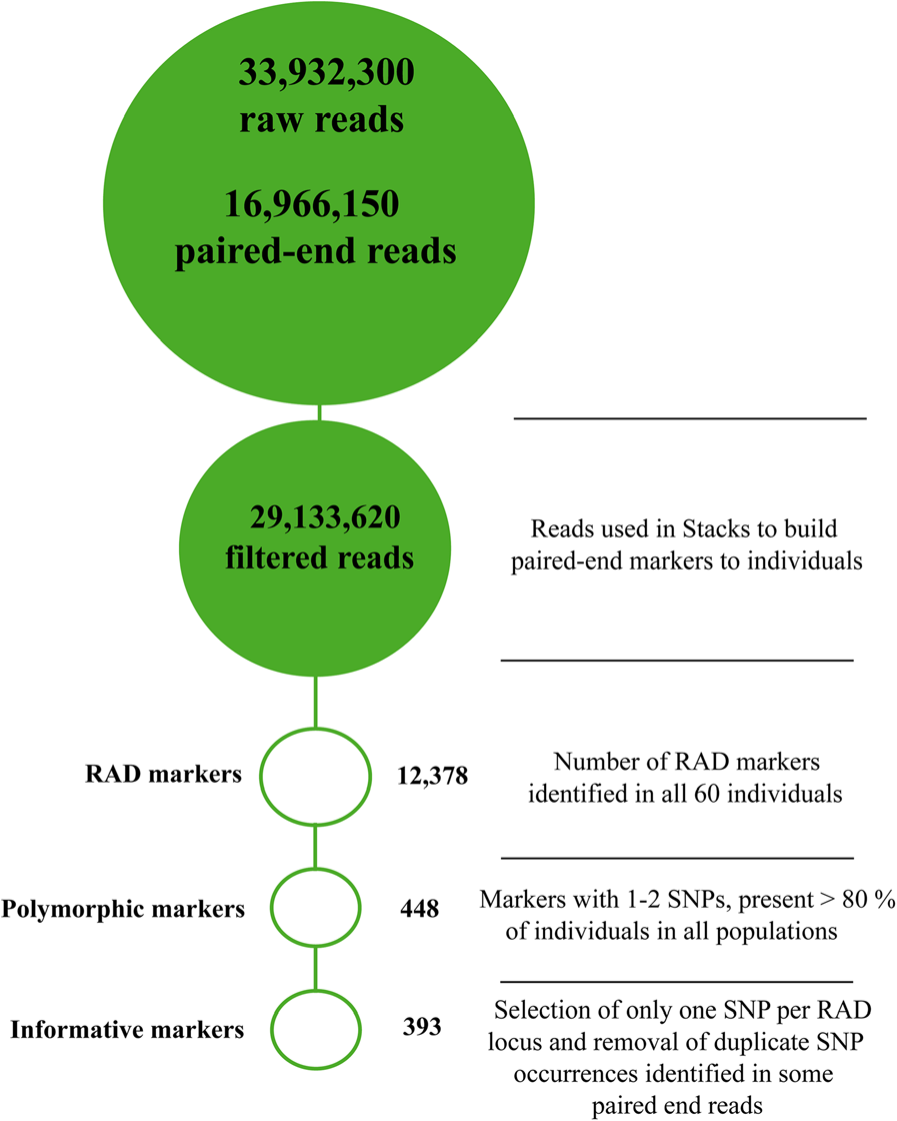
Summary of ddRAD sequencing for *Arapaima gigas*, scheme modified from Brown and collaborators [7]. Workflow of data processing from the obtained raw reads (upper disk) down to the markers used to investigate genomic diversity and structure in populations

The SNP identification from the ddRAD sequencing for 60 individuals of *A. gigas* is summarised in Fig. 1. In total, 33,932,300 raw reads comprising 16,966,150 paired-end reads were obtained from the MiSeq run. After sample demultiplexing and quality filtering, a total of 29,133,620 reads (85.8%) were kept. Assembling loci (RAD-tags) into the 60 individuals identified 12,378 unique RAD-tags. The numbers of RAD-tags and observed heterozygosity per individual are given in Additional file 1. The panel resolved down to 448 markers containing 1–2 SNPs present in more than 80% of individuals in each of the five populations. A further filtering was used to select only one SNP (the most polymorphic one) when two SNPs were identified in a RAD locus, and also removed the few instances where the same SNP was identified at the 3′ end of paired reads. This reduced the panel used for initial population genetic analyses to 393 SNPs (detailed in Additional file 2). Only 2.3% of *A. gigas* ddRAD generated RAD loci (135 bases long) were identified to contain SNPs. This was 3–8 times lower than observed for other teleosts studied at the University of Stirling using the same methodology and analysis (Table 1). The levels of polymorphism detected among the *A. gigas* samples from different locations varied significantly (*P* < 0.05); higher polymorphic levels were found in the Amazon (3.5%) and Solimões (2.7%) rivers compared to the Araguaia river (1.0%), whilst fish from the Tocantins river and the Captive stock showed intermediate levels (2.0%) (*P* < 0.05; Table 2).

**Table 1:** Comparison of basic Stacks [55] statistics for *Arapaima gigas* versus a range of other fish species that were generated using the same ddRAD methodology and Stacks parameters

Species	Common name	Family	*n*° fish	Unique stacks per individual	*n*° Polymorphic loci (%)	SNPs found	Reference
*Arapaima gigas*	Pirarucu	Osteoglossidae	60	6667.9 ± 611.6	154.0 ± 103.8 (2.3) ^F^	192.6 ± 141.4	Present study
*Dicentrarchus labrax*	European seabass	Moronidae	26	6830.9 ± 201.5	519.2 ± 522.1 (7.6) ^E^	1034.7 ± 784.0	[66]
*Cyprinus carpio*	Common carp	Cyprinidae	85	7697.4 ± 1213.4	637.6 ± 416.8 (8.3) ^DE^	868.6 ± 553.9	Unpublished
*Oreochromis niloticus*	Nile tilapia	Cichlidae	6	14,612.8 ± 520.6	1796.2 ± 130.0 (12.3) ^CDE^	2463.3 ± 161.9	[6]
*Clarias anguillaris*	Mudfish	Clariidae	5	9666.0 ± 79.2	1356.6 ± 43.2 (14.0) ^BCD^	1945.4 ± 83.0	Unpublished
*Sprattus sprattus*	European sprat	Clupeidae	8	15,140.6 ± 1561.5	2629.5 ± 319.8 (17.4) ^ABC^	3444.5 ± 441.0	Unpublished
*Ctenolabrus rupestris*	Goldsinny wrasse	Labridae	20	11,817.4 ± 3772.6	2217.9 ± 715.6 (18.8) ^AB^	3129.3 ± 944.5	[67]
*Melanogrammus aeglefinus*	Haddock	Gadidae	16	12,481.8 ± 3255.7	2529.1 ± 415.5 (20.3) ^A^	3349.4 ± 550.2	Unpublished

Results represent the mean values from genotyped individuals. Different letters indicate significant differences (Kruskal-Wallis, *P* < 0.05)

**Table 2:** Comparison of ddRAD Stacks statistics for the different populations of *Arapaima gigas*

Populations	*n*°	Unique stacks per individual	*n*° Polymorphic loci (%)	SNPs found
Amazon	12	6744.3 ± 330.9	241.3 ± 160.2 (3.5) ^AB^	309.4 ± 241.8
Solimões	12	7029.3 ± 397.2	193.5 ± 25.8 (2.7) ^A^	230.1 ± 31.3
Tocantins	12	6582.5 ± 416.6	131.1 ± 21.5 (2.0) ^B^	157.7 ± 24.5
Araguaia	12	5838.9 ± 462.0	60.8 ± 13.1 (1.0) ^C^	80.6 ± 20.3
Captive	12	7144.7 ± 437.0	143.1 ± 26.9 (2.0) ^B^	185.2 ± 33.2

Results represent the mean values from genotyped individuals. Different letters indicate significant differences (Kruskal-Wallis, *P* < 0.05)

### Analyses of genetic diversity

**Fig. 2:**
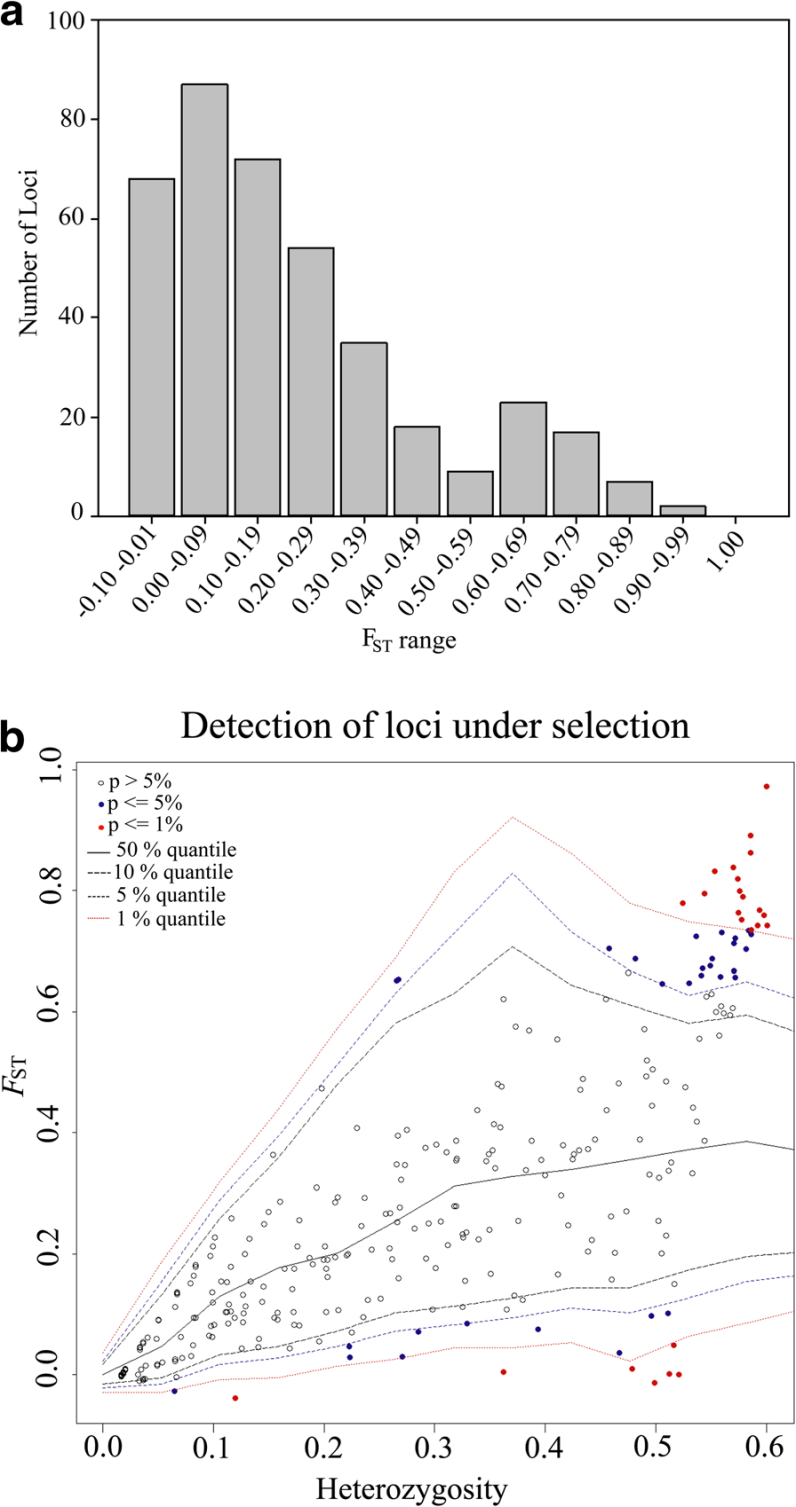
Loci analysis for 392 SNPs across 60 individuals analysed from the five populations of *Arapaima gigas*. **a** Frequency distribution of *F*_ST_ values. **b** Detection of loci under selection (outlier) using hierarchical structure model implemented in Arlequin v. 3.5.2.2. Outlier loci are indicated in red (*P* < 0.01) and blue (*P* < 0.05) dots

The 393 loci analysed were all found to be in HWE both within individual population groupings and across the global population sample (*P* < 0.05; sequential Bonferroni α = 0.05/393 corrected). Analyses of LD indicated significant association between locus 373_82 and 3408_78 in the global dataset after Bonferroni correction (α = 0.05/61463). Therefore, locus 373_82 (lower *F*_ST_) was removed from further analyses resulting in a final dataset with 392 SNPs. For this dataset (392 SNPs, 60 individuals), the global *F*_ST_ calculated across all loci was 0.389, and individual locus *F*_ST_ values ranged from − 0.04 to 0.97 with their frequency distribution depicted in Fig. 2a. Analysis also detected 57 loci putatively under selection (outlier) (Fig. 2b). Outlier SNPs were kept in the dataset in order to integrate possible adaptive information in further population analysis [44], while their removal did not significantly alter population structure results (data not shown).

All pairwise *F*_ST_ comparisons between population samples were significant (*P* < 0.05; Bonferroni correction α = 0.05/10; Table 3). Moderate degrees of differentiation were found between the Amazon and Solimões (0.086) populations and between the Captive and Araguaia (0.077) populations. High degrees of differentiation were found between Tocantins and Captive (0.227), Tocantins and Araguaia (0.332), Solimões and Tocantins (0.336), Amazon and Tocantins (0.390), Solimões and Captive (0.423) and Amazon and Captive (0.459) population samples. The highest levels of genetic differentiation were observed between Araguaia and two other samples (Amazon (0.556) and Solimões (0.523)). Table 4 also indicates waterway distances calculated among sampling sites, and Mantel test based on these values indicated that the isolation by distance hypothesis was not supported (*P* = 0.104; R^2^ = 0.65).

**Table 3:** Genetic differentiation (*F*_ST_) and geographical distance (km) among populations of *Arapaima gigas*

	Population pairwise *F*_ST_ / Geographical distance (km)				
	Amazon	Solimões	Tocantins	Araguaia	Captive
Amazon	–	1323	3613	4708	–
Solimões	0.086*	–	2290	3385	–
Tocantins	0.390*	0.336*	–	1095	–
Araguaia	0.556*	0.523*	0.332*	–	–
Captive	0.459*	0.423*	0.227*	0.077*	–

Below diagonal values are pairwise *F*_ST_ comparisons made with Arlequin v. 3.5.2.2, performing 10,000 permutations. Above diagonal values depict waterway geographical distance measured among wild populations (*Captive* excluded) using Google Earth version 7.1.8 (https://www.google.com/earth). * *P* < 0.05; Bonferroni correction α = 0.05/10)

**Table 4:** Genetic diversity statistics for the five population samples of *Arapaima gigas*

Populations	Fish (*n*°)	Polymorphic loci (%)	Private alleles (*n*°)	I	H_O_ ± SE	H_E_ ± SE	*F*_IS_ ± SE
Amazon	12	71.4	62	0.316 ± 0.013	0.192 ± 0.010	0.203 ± 0.009	0.034 ± 0.016
Solimões	12	70.4	49	0.316 ± 0.013	0.220 ± 0.011	0.204 ± 0.009	− 0.070 ± 0.013
Tocantins	12	39.0	9	0.190 ± 0.013	0.134 ± 0.011	0.125 ± 0.009	− 0.061 ± 0.016
Araguaia	12	14.5	9	0.061 ± 0.008	0.042 ± 0.007	0.039 ± 0.006	−0.073 ± 0.013
Captive	12	34.7	5	0.148 ± 0.011	0.110 ± 0.009	0.092 ± 0.007	− 0.174 ± 0.010
Overall	60	46.0 ± 10.9	**–**	0.206 ± 0.006	0.140 ± 0.005	0.133 ± 0.004	−0.052 ± 0.007

Polymorphic loci (%), *I* Shannon’s Information Index, *H*_*O*_ observed heterozygosity, *H*_*E*_ expected heterozygosity, *F*_IS_ coefficient of inbreeding of Weir and Cockerham [61], *SE* standard error. Indices calculated using 392 SNPs with GenAlEx v. 6.5

**Fig. 3:**
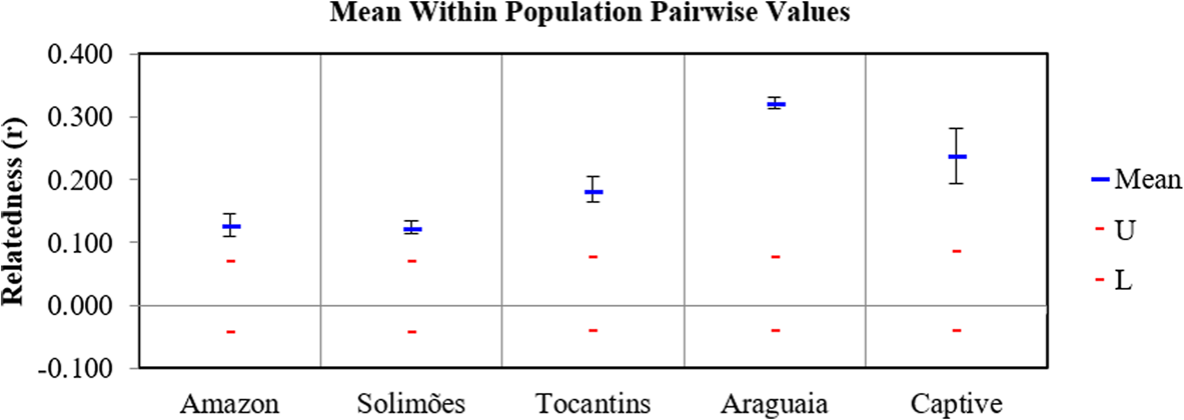
Within population pairwise mean relatedness (r) for *Arapaima gigas* (*n* = 12 individuals for each population). Calculations followed method of Lynch and Ritland [62] with confidence intervals of 95% denoted by the U (upper) and L (lower) marks, calculated after 1000 bootstrap resamplings and 1000 permutations in GenAlEx version 6.5

Overall, the population samples from the Amazon basin (Amazon and Solimões) were genetically more diverse than those from the Araguaia and Tocantins rivers in terms of percentage of polymorphic loci, number of private alleles, Shannon’s Information Index (I) and observed heterozygosity (H_O_) (Table 4). The mean pairwise relatedness (r) between individuals was significantly different from zero for all population samples (*P* < 0.05), with lower r from the rivers Amazon (0.127) and Solimões (0.125), increased r from Tocantins (0.182) and the r highest from Araguaia (0.323) and the Captive population (0.238) (Fig. 3). Relatedness correlated negatively with H_O_ and with I (R^2^ = 0.957, *P* < 0.01, and R^2^ = 0.956, *P* < 0.01, respectively), indicating inbreeding as a potential cause for the loss of genetic diversity in the studied populations.

### Population structure

**Fig. 4:**
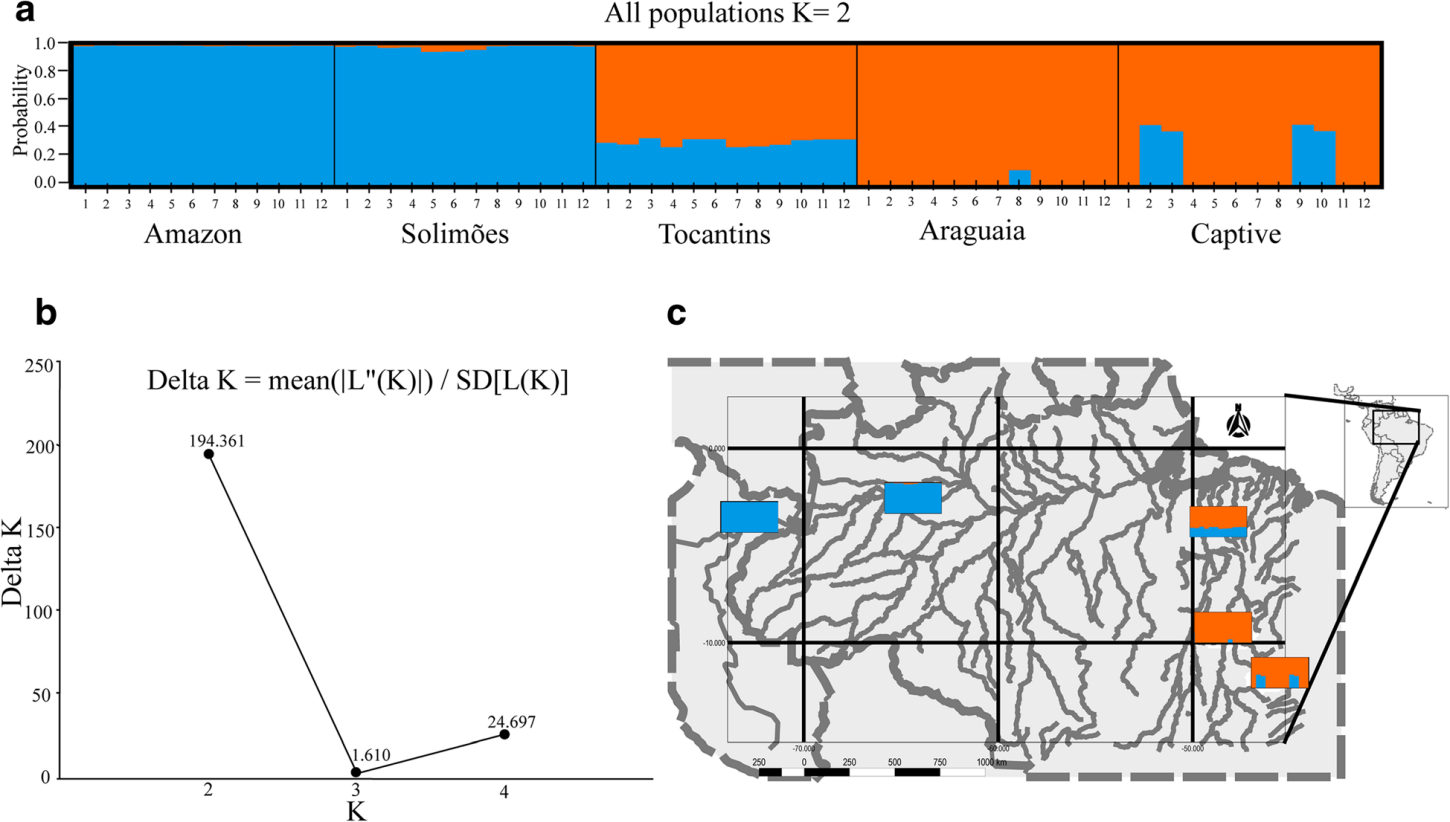
Bayesian clustering representation for populations of *Arapaima gigas* using 392 SNP markers in STRUCTURE v. 2.3.4 [63]. **a** Analysis of five populations (*n* = 60 individuals; optimal Evanno’s K = 2). **b** Graphical representation of optimal number of clusters (K) across the five studied populations determined by Evanno’s method, where highest Delta K indicate the real number of populations [64], indicated by delta K peaking at K = 2. **c** Geographical representation of structure results for the global population

The number of clusters (K) across the five studied population samples was resolved by the Evanno method, to be two (Fig. 4b). Analysis suggested the Amazon river basin (Amazon and Solimões) and Araguaia river are distinct genetic stocks, and suggested the lower Tocantins river sample is a hybrid zone between these two groupings (Fig. 4a–c). Analyses also showed eight individuals from the Captive population to be genetically similar to the Araguaia population, and four individuals similar to the Tocantins population (Fig. 4a).

The DAPC analysis identified three groups (K = 3) and agreed with the findings resolved by STRUCTURE. Populations from the Amazon basin (Amazon and Solimões) were clustered together, the genetically “hybrid” area formed by Tocantins was considered as a distinct cluster in DAPC, which also included four Captive individuals. The third cluster grouped together Araguaia and the remaining eight Captive individuals (Additional file 3A). Selection of three clusters was suggested by the Bayesian Inference Criterion (BIC) (decreasing elbow; Additional file 3B), with all individuals being correctly reassigned to their original clusters with 100% membership probability (Additional file 3C).

## Discussion

Comparatively few studies, all of limited scope, have been undertaken to characterise genetic variability in the iconic *A. gigas* [29–31, 37–40]. All have been restricted by the relatively low resolution provided by the genetic markers available for use (i.e. either a single mitochondrial locus, 14 variable microsatellites or dominant AFLP markers). The large 393 SNP set surveyed in the current study provides a potential step change in resolution available to resolve genetic diversity within and among *A. gigas* populations, allowing a robust genome wide snapshot of variability to be quantified [4, 45]. The screened SNP markers were found to be in HWE with only two loci found to be statistically associated (LD). Further population analyses identified 335 neutral and 57 potential outlier loci. Putative outlier loci were incorporated in the dataset as they can provide valuable information on local adaptation and their inclusion is recommended in analysis of threatened species for aiding define conservation units [44].

The most striking finding of the survey was the extremely low level of genetic variability resolved, both within and between individuals from each of the wild population samples, overall being 3–8 times lower than comparable values for other fish species studied using the same methodology. A low level of detectable variability has also been reported for Northern pike (*Esox lucius*) [46] where very few SNPs were identified (c. 1% of RAD loci being polymorphic) using a RAD based methodology. Similarly, very low levels of polymorphism in populations of other fish species have been reported elsewhere, e.g. brown trout *Salmo trutta* [47], the estuarine black bream *Acanthopagrus butcheri* [48] and the shark *Carcharhinus plumbeus* [49]. Samples from the Amazon, Solimões and Tocantins rivers were caught by individual fishermen which, given the solitary behaviour of adult *A. gigas* in the Amazon floodplains, is likely to have minimised sampling bias [29, 31]. However, the sample from the Araguaia river was obtained during a rescue operation, when juvenile fish were trapped in a single small lagoon during the dry season and likely explains the particularly high genetic relatedness (0.323) recorded for this sample. It is likely that the observed lack of genetic variability within the *A. gigas* populations studied has been influenced by past bottleneck effects, both historic and those more recently documented [22, 32], while genetic drift may have accentuated genetic differences between rivers and regions. The analysis of polymorphism levels in pristine populations from more remote areas could help resolve this issue.

Population samples from the Amazon and Solimões rivers were more genetically variable (percentage of polymorphic loci, number of private alleles, Shannon’s index and observed heterozygosity) compared to fish from the Araguaia. Interestingly, samples from the river Tocantins and the captive stock showed intermediate diversity levels. These observations confirm and complement previous studies using mtDNA and microsatellite markers which indicated higher diversity in *A. gigas* sampled from the Amazon and Solimões compared to the river Tocantins [31, 38], and very low levels of genetic diversity in samples from the Araguaia river [30, 40]. The higher genetic diversity in populations from the Amazon basin has previously been suggested to be a consequence of the larger population size in these less urbanised environments. The increased severity of the droughts during the dry season in the Araguaia river [30], may also be a causal factor associated with lower genetic diversity in *A. gigas* in this drainage.

Importantly, all five population samples surveyed showed significant levels of relatedness (r). Mean population relatedness was negatively correlated with diversity indexes such as observed heterozygosity (H_O_) and Shannon’s Index, which is indicative of a degree of inbreeding, similar to that reported for mudminnows (*Umbra krameri*) [50]. An elevated degree of relatedness can be characteristic of populations restocked with related individuals [50]. Populations of *A. gigas* have been overexploited for more than 200 years, leading to reduced numbers (only 13% of original population estimates), and even extinction in many localities [31, 32]. Restocking with related individuals has been a common practice in some of these areas [32, 51, 52]. However, while high levels of relatedness were observed within each population studied, *F*_ST_ pairwise comparisons showed clear differentiation among populations. This mirrors findings from other genetic studies of *A. gigas* populations from Amazon, Tocantins and Araguaia rivers [29–31, 40], where past bottleneck events were considered to be the main factor underlying current population genetic structure [30, 31]. The relatively sedentary behaviour of *A. gigas* has been regarded as a major factor contributing to low genetic flow, resulting in local population differentiation [29, 40]. Ecological studies using radio-telemetry to monitor wild individuals also found strong patterns of residency and territoriality in *A. gigas* [53]. Indeed, a study evaluating the dispersal capacity of *A. gigas* concluded the existence of high levels of genetic similarity among lakes separated by 25 km, moderate genetic differentiation in sites separated by 100 km and highest genetic differentiation among regions separated by > 1500 km [29], supporting previous ecological observations. In the current study, the high inter-population differentiation together with the lack of support for isolation by distance and the elevated levels of mean population relatedness negatively correlated with diversity (I and H_O_), corroborate the hypothesis that genetic drift led to a loss of genetic variability and increased differentiation between populations of *A. gigas* [31]. Analyses of the captive population also illustrated aquaculture playing a role in fish translocation, with the studied broodstock having clearly multiple geographical origins.Translocation of arapaima for aquaculture development has been considered a key issue for the species conservation [22], and the developed SNP panel will greatly assist broodstock identification and pedigree management, prerequisites for the rational maintenance and monitoring of stock genetic diversity.

An initial population genetic study of *A. gigas* within the Amazon basin, using two discontinuous mitochondrial DNA regions of 1204 base-pairs (bp) (NADH1 segment) and 1143 bp (ATPase segment) revealed minimal evidence of substructuring, which loosely fitted an isolation-by-distance model [31]. A later study, based on seven microsatellite markers and using Bayesian analysis in STRUCTURE detected two distinct clusters, one comprising fish mostly from the lower Tocantins and lower Amazon rivers, the other comprising *A. gigas* predominantly from the mid- region of the Amazon river [29]. A more recent study of population sampled from the Essequibo river basin using 11 hypervariable microsatellite markers and mtDNA markers (NADH1 segment), identified patterns of allopatric differentiation within the species which led the authors to suggest that sympatric “species” could be present in three of the sampled sites [37]. In support of this finding, it was suggested that *Arapaima* belongs to a multispecies group based on morphological analyses [35, 36]. The current SNP-based analysis contrasts with these previous findings as it identified significant substructuring both within and between river basins. Though the data did not fit an isolation by distance model, this cannot be ruled out as only a few populations were available for analysis. Despite some high pairwise *F*_ST_ values, > 96% of the RAD loci identified as polymorphic contained only one or two SNPs. This is a much higher proportion than that observed for a range of other fish species using a comparable methodology. A Stacks-based analysis of crypto species would be expected to reveal higher levels of polymorphisms both among and within RADtags. Therefore, the present genetic data do not provide supportive evidence for genetically distinct species within the samples and populations analysed.

Clearly, evidence regarding the overall low levels of variability within populations and significant genetic structuring among populations should guide future management and conservation plans for wild *A. gigas*. Retaining as much diversity as possible should be a priority, and this can only be realistically achieved through coordinated conservation efforts over a broad geographic scale, and should be informed by regular genetic monitoring. Similarly, the findings of this study have specific implications for the development of commercial *A. gigas* aquaculture. A concern in all aquaculture enterprises, where high fecundity is the norm, is the potential for deleterious inbreeding. This will require particular awareness and careful genetic management for *A. gigas*. Also, given the low genetic diversity baseline, the potential for commercial traits gain through selective breeding may be limited. While now being widely employed for improvement of many aquaculture species such as the Nile tilapia (*Oreochromis niloticus*), the Atlantic salmon (*Salmo salar*), the rainbow trout (*Oncorhynchus mykiss*) and several others, reviewed by Yue [9], the application of genomic selection is also likely to be much more challenging for *A. gigas*, both in terms of marker development and number of pedigrees required to collect relevant data. It may be prudent to consider establishing farm strains from a wide range of wild populations, which would maximise genetic diversity and also act as gene banks for the species.

## Conclusions

Though restricted by the unusually low level of polymorphism detected within the arapaima genome, the screening of hundreds of SNPs using ddRAD technology can be considered as a reliable and robust method to determine genetic variability within and between arapaima populations. Furthermore, these genetic markers have a role to play in identifying the origin of animals used in aquaculture, and in informing the maintenance of the genetic diversity of captive reared broodstocks. There is also the opportunity to develop a subset of the most informative SNPs for screening using alternative platforms (qPCR assays; small scale SNP chips), which require less labour intensive and less expensive protocols. Such a panel would probably be better suited than RAD for the much more extensive survey of arapaima populations that is required to better understand and manage the species. As a priority, surveys should focus on the different morphotypes of *A. gigas* (orange-fleshed and white-fleshed) and the contended species recently described for *Arapaima* [35, 36] which currently lacks support from molecular data. More detailed genetic studies (i.e. genome scans, QTL analyses, linkage mapping) need much more dense marker panels. While this would be feasible using RAD approaches in arapaima, it is clear that it would require the selection of restriction enzymes that cut Arapaima DNA much more frequently, which would necessitate a 10–50 fold higher sequencing effort than currently used, with associated budget implications.

## Methods

### Arapaima samples

**Fig. 5:**
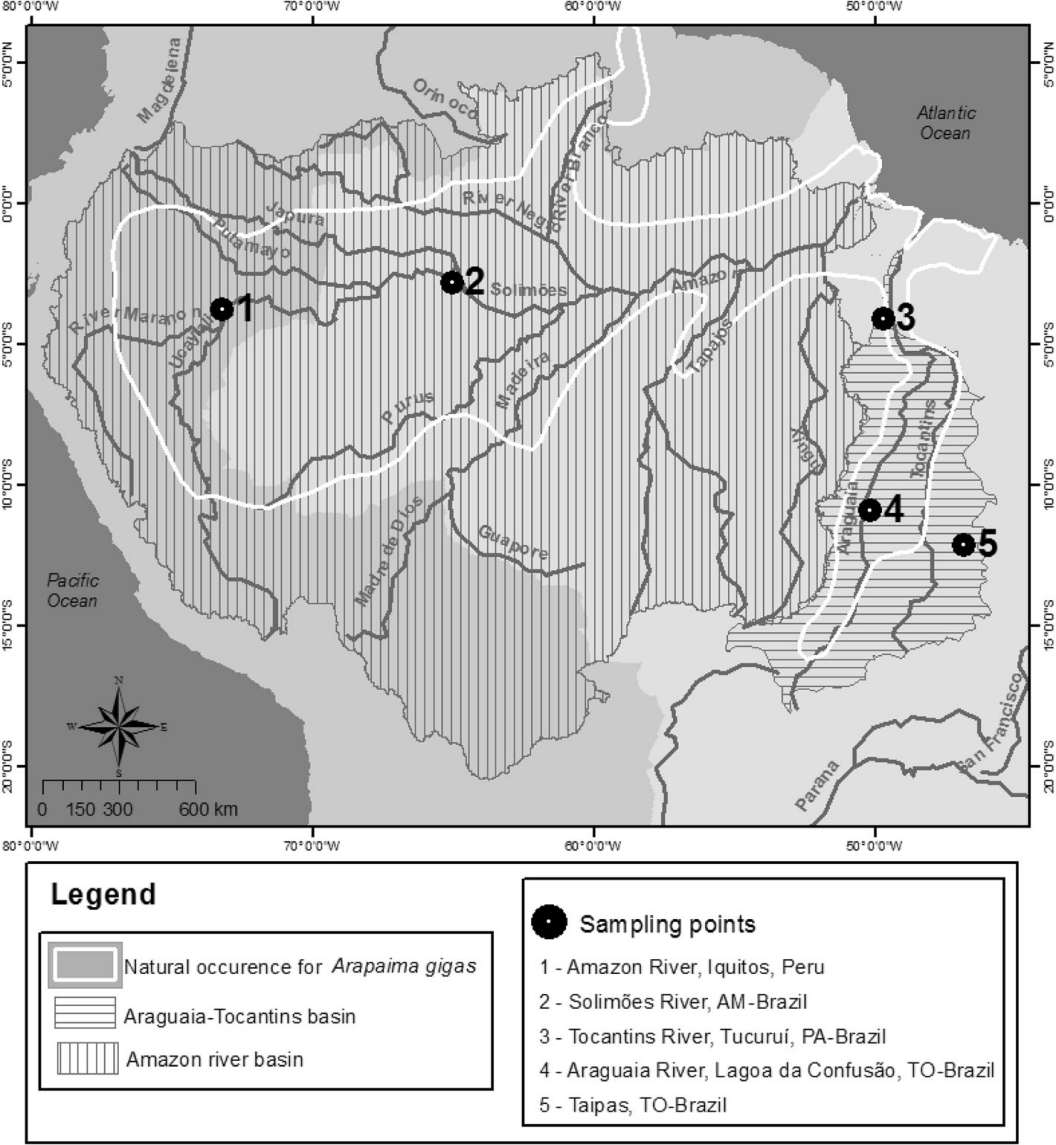
Map of Amazon region showing the natural distribution of *Arapaima gigas* according to Castello and Stewart [22] (*by* Marta E. Ummus using software ArcGIS). Sampling points include two wild populations from Amazon river Basin (Amazon - 1 and Solimões - 2), two wild populations from Tocantins-Araguaia Basin (Tocantins – 3 and Araguaia - 4), and one captive population (Captive - 5) from Tocantins state, Brazil

This study analysed samples from the rivers Amazon (Iquitos, Perú), Solimões (Jarauá, AM, Brazil), Tocantins (Tucuruí, PA, Brazil), Araguaia (Lagoa da Confusão, TO, Brazil), and also a captive broodstock (Taipas, TO, Brazil). Twelve individuals per population / stock were randomly selected from a wider number of sampled specimens, giving a total of 60 individuals (Fig. 5). Samples from Amazon, Solimões and Tocantins rivers were collected from wild captured animals harvested by fisherman during the legal fishing season, samples from these fish having been previously studied using other molecular markers [29, 31]. Animals from Araguaia were opportunistically sampled during a rescue operation where juvenile fish that were trapped in a small lagoon were sampled and later released into an adjacent larger lagoon. The Captive broodstock comprised adults of unknown hydrographic origin, filial generation or degree of relatedness, and were sampled from a farm located in Taipas-TO, Brazil. Fin clips or muscle samples were fixed in 95% ethanol and kept at − 20 °C until DNA extraction at the University of Stirling (Scotland-UK). Table 5 includes information on geographical coordinates and total number of originally sampled fish in each location. All samples were collected in accordance with Brazilian regulations (process number 02001.007554/2005-76 IBAMA/MMA).

**Table 5:** Studied populations of *Arapaima gigas*

Site name	*n*° genotyped fish	*n*° sampled fish	Sampling year	Location	Coordinates
Amazon	12	16 [31]	2000	Amazon River, Iquitos, Peru	−3.767454/−73.248425
Solimões	12	223 [29]	2003	Solimões River, Tefé, AM-Brazil	−2.807539/−65.076747
Tocantins	12	38 [29]	2002	Tocantins River, Tucuruí, PA-Brazil	−4.111435/− 49.744085
Araguaia	12	31	2013	Araguaia River, Lagoa da Confusão, TO-Brazil	−10.918678/−50.183229
Captive	12	24	2016	Taipas, TO-Brazil	−12.161331/−46.859444

Information on site locations and geographical coordinates for one captive (Captive) and four wild (Amazon, Solimões, Tocantins and Araguaia) populations. A reference is indicated where samples were previously analysed in another study

### DNA extraction

For genomic DNA extraction, a fin clip or muscle sample from each of the 60 individuals was initially incubated for 16 h at 55 °C in a lysis solution containing 200 μL of SSTNE buffer [54], 20 μL 10% SDS, 5 μL proteinase K (10 mg.mL^− 1^). The temperature was then increased to 70 °C for 15 min to inactivate the proteinase K, before 5 μL RNAse A (2 mg.mL^− 1^) was added to each sample and further incubated at 37 °C for 1 h. To precipitate proteins, 160 μL 5 M NaCl (0.7 volumes) was then added, mixed and the tubes left on ice for 10 min. This mixture was centrifuged at 22,000 rcf for 10 min to pellet the proteins. A proportion of the supernatant (c. 300 μL) was then transferred to a new microfuge tube containing an equal volume of isopropanol, mixed to precipitate DNA and centrifuged at 22,000 rcf for 1 min to pellet the DNA. The supernatant was carefully removed and the DNA pellet was then washed in 1 mL 70% ethanol for 3 h, after which the 70% ethanol was replaced and the pellet washed for a further 16–20 h. Finally, the samples were centrifuged at 22,000 rcf for 5 min, the ethanol was discarded and the DNA pellet air dried and reconstituted over a 24-h period in 5 mM Tris (pH 8.0). The DNA quality and concentration were evaluated by spectrometry (NanoDrop; Thermo Scientific, USA). Agarose gel electrophoresis (0.8%) was used to check the integrity of the genomic DNA. Finally, DNA concentration prior to library preparation was more accurately quantified by fluorescence assay (Qubit 2.0, Thermo Scientific, USA).

### Library preparation and sequencing

A ddRAD library was prepared according to a published method [19] while implementing minor modifications detailed in full in Brown and collaborators [7]. Duplicate restriction digestion reactions were undertaken for each arapaima DNA sample, with different barcoded adapter combinations being used for each restriction digestion. Following sequencing, the duplicate data sets for individuals were combined. Briefly, for each reaction 15 ng DNA was co-digested at 37 °C for 30 min with 0.3 U *Sbf*I (‘rare’ cutter, CCTGCA|GG motif) and 0.3 U *Sph*I (‘common’ cutter, GCATG|C motif) high fidelity restriction enzymes (New England Biolabs; NEB) in a 5 μL reaction volume that included 1× CutSmart™ buffer (NEB). After cooling the reactions to room temperature, 2.5 μL of a premade barcode-adapter mix was added to the digested DNA, and incubated at room temperature for 10 min. This adapter mix comprised individual-specific barcoded combinations of P1 (*Sbf*I-compatible) and P2 (*Sph*I-compatible) adapters at 6 and 72 nM concentrations respectively, in 1× reaction buffer 2 (NEB). Adapters were compatible with Illumina sequencing chemistry. The barcoded adapters were designed such that adapter–genomic DNA ligations did not reconstitute RE sites, while residual RE activity limited concatemerization of genomic fragments during ligation. The adapters included an inline five- or seven-base barcode for sample identification. Ligation was performed over 75 min at 22 °C by addition of a further 2.5 μL of a ligation mix comprising 4 mM rATP (Promega, UK), and 2000 cohesive-end units of T4 ligase (NEB) in 1× CutSmart buffer. The reactions were terminated by addition of 20 μL PB buffer (Minelute PCR Purification Kit; Qiagen UK). Each DNA sample was processed in duplicate (i.e. 120 separate digestion – ligation reactions). All 120 reactions were pooled and column-purified (MinElute PCR Purification Kit) and eluted in 52 μL of EB buffer (Qiagen, UK). The purified pooled sample was separated by agarose gel (1.1%) electrophoresis and fragments ranging from approximately 450 to 650 bp were excised and purified using a MinElute Gel Extraction Kit (Qiagen, UK). The eluted size-selected template DNA (c. 54 μL in EB buffer) was amplified by PCR (11 cycles; 20 separate 20-μL reactions, each with 1.5 μL of pooled template DNA) using Q5 Hot Start High-Fidelity DNA Polymerase (NEB) and Illumina compatible primers. The PCR reactions were combined (400 μL total) and column-purified with the MinElute PCR Purification Kit (Qiagen, UK), then eluted in 50 μL of EB buffer. The library was then re-purified, to ensure no carry over of PCR primer dimer or other low size range amplicons (< 200 bp) using an equal volume of AMPure magnetic beads (Perkin-Elmer, UK) and eluted in 15 μL EB buffer. Finally, the library was sequenced at the Institute of Aquaculture (University of Stirling, Scotland) on a single run on the Illumina MiSeq platform (162 bp paired end reads, 300 cycle kit, v2 chemistry kit; Illumina, Cambridge, UK).

### ddRAD genotyping and comparative datasets

After sequencing, reads were de-multiplexed, low quality reads with missing restriction sites and containing ambiguous barcodes were excluded and sequences trimmed to 135 bases long using Stacks version 1.42 [55]. Given the lack of a reference genome for *A. gigas*, the RAD loci were assembled de novo using the following parameter settings: 4 as the minimum read depth to create a stack (m), 2 as the maximum number of mismatches in an individual (M) and 1 as the maximum mismatch between loci for building the catalogue (*n*). A robust set of SNPs suitable for population analyses was exported from the catalogue in Genepop format [56], using the ‘populations’ module within Stacks to select only those SNPs that were scored in at least 80% of individuals in each of the five populations and confined to RAD-tags that contained no more than two SNPs.

The levels of polymorphism within the *A. gigas* samples were compared to ddRAD data of other teleosts generated from other projects undertaken at the University of Stirling using the same methodology. These species and numbers of samples were: *Cyprinus carpio* L. (*n* = 85), *Dicentrarchus labrax* (L.) (*n* = 26), *Oreochromis niloticus* (L.) (*n* = 6), *Clarias anguillaris* (L.) (*n* = 5), *Sprattus sprattus* (L.) (*n* = 8), *Ctenolabrus rupestris* (L.) (*n* = 20) and *Melanogrammus aeglefinus* (L.) (*n* = 16). For each species, the mean (± SD) of the following parameters were calculated: number of unique stacks, number of polymorphic loci and number of SNPs obtained. Non-parametric Kruskal–Wallis one-way ANOVA followed by Dunn’s pairwise post hoc tests were used to compare the ratio between polymorphic loci and unique stacks obtained between species. Statistical analyses were conducted using Minitab version 17.3.1 (Minitab, PA, USA) with significance set at *P* < 0.05.

### Analyses of genetic diversity and structure

Initially, tests for Hardy-Weinberg Equilibrium (HWE) and linkage disequilibrium (LD) were conducted for the SNP loci both within populations and across the global dataset (60 individuals, five populations) using Genepop version 4.6 [56]. To do so, the Markov chain Monte Carlo (MCMC) parameters used were 10,000 dememorizations, 20 batches and 5000 iterations per batch). The software Arlequin version 3.5.2.2 [57] was used to estimate *F*_ST_ values for each locus in the global population, to estimate pairwise genetic differentiation (*F*_ST_) between populations (10,000 permutations; *P* < 0.01) and to identify outlier loci (hierarchical island model, 20,000 simulations, 100 demes simulated per group with 10 groups simulated). Sequential Bonferroni [58] corrections for type I errors were applied when multiple tests were performed.

To investigate the hypothesis of isolation by distance, the shortest waterway path among sampling sites (Captive excluded) was measured using Google Earth version 7.1.8 (https://www.google.com/earth). The software GenAlEx version 6.5 [59, 60] was used to perform a Mantel test in which geographical (km) and genetic (*F*_ST_) distances were correlated. GenAlEx was also used to estimate percentage of polymorphic loci, number of private alleles, Shannon’s Information Index (I), expected (H_E_) and (H_O_) observed heterozygosity, and coefficient of inbreeding (*F*_IS_) from Weir and Cockerham [61]. In GenAlEx, pairwise individual relatedness was estimated using a method published by Lynch and Ritland [62] to calculate a square matrix of individual pairwise relatedness (r). The average of pairwise values was calculated using the “Pops mean” option, in which significance was tested using 1000 permutations and 1000 bootstrap resamplings to estimate 95% confidence intervals. Pearson Product Moment Correlations was used to correlate levels of relatedness (r) with Shannon’s I and H_O_ using Minitab (*P* < 0.01).

Population structure was first investigated using a model-based approach, which assumes HWE and linkage equilibrium, implemented in STRUCTURE version 2.3.4 [63]. Initially, estimation of the K-value which maximizes the global likelihood of the dataset (50,000 burn-in; 100,000 MCMC; 10 independent runs *per* K; ranging K from 1 to 5) was made using an admixture model and frequencies were assumed correlated. Optimal K-value was determined by Evanno’s method [64] using the Best K pipeline of CLUMPAK program [65]. Using the best K-value, a final analysis was conducted with a burn-in of 250,000 using 500,000 MCMC and 10 replications. Results were then averaged and displayed using main pipeline of CLUMPAK. The analysis described above was independently conducted for the global dataset (5 populations), and then for the Amazon river basin (Amazon and Solimões) and the Tocantins-Araguaia river basin (Tocantins, Araguaia and Captive), separately.

Finally, population structure was explored using a discriminant analysis of principal components (DAPC), conducted in R version 3.3.2. using the package Adegenet v. 2.0.1. DAPC analysis is not a model-based approach, and it optimizes the variance between groups while minimizing the differences within clusters. It requires prior identification of a cluster number, which was made using the Bayesian Information Criterion (BIC). The function *find.clusters* was used to transform original data into principal components (PC), retaining 60 PCs in the analysis. The *dapc* function performed a discriminant analysis using 20 PCs (> 80% of variance explained) and 5 eigenvalues were retained and examined. The *assign.per.pop* function was used to evaluate the proportions of successful reassignment of individuals to their original clusters.

## Additional files

Additional file 1: General information and summary data from the ddRAD study of *Arapaima gigas*: a) the combinatorial inline barcodes used for each individual - DNA samples were processed in duplicate, with a different barcode combination used for each RE digestion - ligation reaction, thus each individual is represented by two sets of identifiers; b) basic RAD locus statistics generated by STACKS for each of the 60 samples; c) mean observed heterozygosity within the 392 loci surveyed for each individual. (XLSX 14 kb)

Additional file 2: Sequence information and associated basic genetic statistics for each of the 393 SNPs used in this study. The position of assayed SNP is indicated by red font within each 135 base RADTag sequence, together with basic genetic statistics (computed with GenAlEx) for the combined sample set (*n* = 60). (XLSX 60 kb)

Additional file 3: A. Discriminant analysis of principal components (DAPC) using 392 SNP markers in *Adegenet* v. 2.0.1 [68] for the five *Arapaima gigas* populations sampled (*n* = 60 individuals). B. Selection of number of clusters was based on Bayesian Inference Criterion (BIC), which indicates 3 clusters for data summarization (elbow drop). C. Membership probabilities (red = 1, white = 0) for individuals into clusters, blue crosses indicate the prior cluster provided into DAPC. (TIF 2254 kb)

## Abbreviations

BIC: Bayesian Inference Criterion
bp: base pair
CITES: Convention on International Trade of Endangered Species of Wild Fauna and Flora
DAPC: Discriminant analysis of principal components
ddRAD: double digest restriction-site-associated DNA
F_IS_: Coefficient of inbreeding
F_ST_: Fixation index
H_E_: Expected heterozygosity
H_O_: Observed heterozygosity
HWE: Hardy-Weinberg Equilibrium
I: Shannon’s Information Index
ISSR: Inter simple sequence repeats
LD: Linkage disequilibrium
MCMC: Markov chain Monte Carlo
mtDNA: mitochondrial DNA
NGS: Next generation sequencing
PC: Principal components
PCR: Polymerase chain reaction
qPCR: quantitative PCR
QTL: Quantitative trait loci
RAD: Restriction-site-associated DNA
SNP: Single nucleotide polymorphism
