# Supplementary figures and images for: Genetic diversity and structure in Arapaima gigas populations from Amazon and Araguaia-Tocantins river basins
Source: BMC Genet. 2019 Jan 28;20:13. doi: 10.1186/s12863-018-0711-y (PMC6348655; doi:10.1186/s12863-018-0711-y)

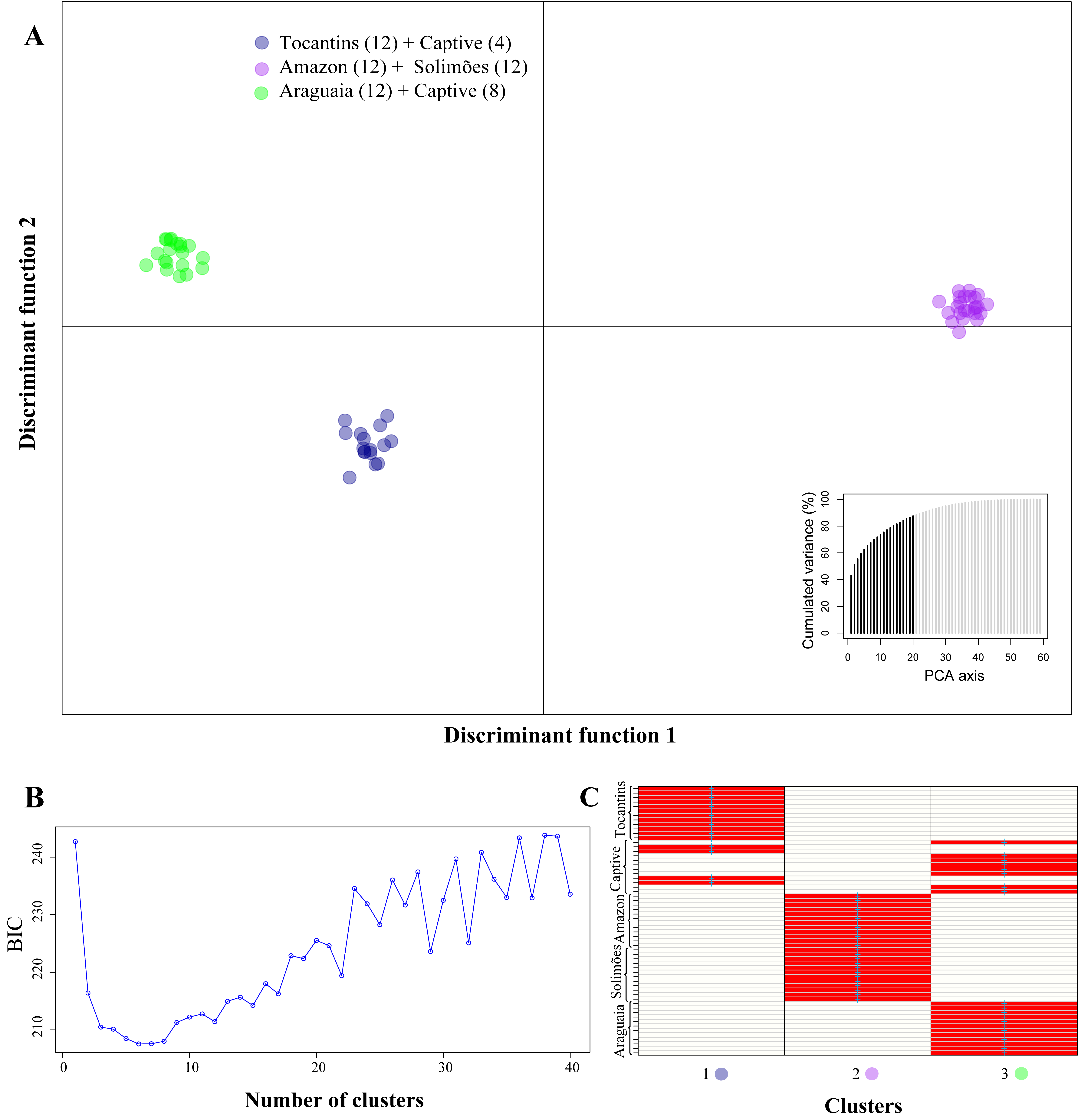

Supplement: Supplementary file 3 — A. Discriminant analysis of principal components (DAPC) using 392 SNP markers in Adegenet v. 2.0.1 [68] for the five Arapaima gigas populations sampled (n = 60 individuals). B. Selection of number of clusters was based on Bayesian Inference Criterion (BIC), which indicates 3 clusters for data summarization (elbow drop). C. Membership probabilities (red = 1, white = 0) for individuals into clusters, blue crosses indicate the prior cluster provided into DAPC. (TIF 2254 kb) [file 12863_2018_711_MOESM3_ESM.tif]
